# Elevated virulence of an emerging viral genotype as a driver of honeybee loss

**DOI:** 10.1098/rspb.2016.0811

**Published:** 2016-06-29

**Authors:** Dino P. McMahon, Myrsini E. Natsopoulou, Vincent Doublet, Matthias Fürst, Silvio Weging, Mark J. F. Brown, Andreas Gogol-Döring, Robert J. Paxton

**Affiliations:** 1 School of Biological Sciences, MBC, Queen's University Belfast, Belfast BT9 7BL, UK; 2 Institute of Biology, Free University Berlin, Schwendenerstrasse 1, 14195 Berlin, Germany; 3 Department for Materials and Environment, BAM Federal Institute for Materials Research and Testing, Unter den Eichen 87, 12205 Berlin, Germany; 4 Institute for Biology, Martin Luther University Halle-Wittenberg, Hoher Weg 8, 06120 Halle (Saale), Germany; 5 German Centre for Integrative Biodiversity Research (iDiv) Halle-Jena-Leipzig, Deutscher Platz 5e, 04103 Leipzig, Germany; 6 School of Biological Sciences, Royal Holloway University of London, Egham, Surrey TW20 OEX, UK; 7 IST Austria (Institute of Science and Technology Austria), 3400 Klosterneuburg, Austria; 8 Institute of Computer Science, Martin Luther University Halle-Wittenberg, 06099 Halle (Saale), Germany

**Keywords:** virulence, emerging infectious disease, pollinator, decline

## Abstract

Emerging infectious diseases (EIDs) have contributed significantly to the current biodiversity crisis, leading to widespread epidemics and population loss. Owing to genetic variation in pathogen virulence, a complete understanding of species decline requires the accurate identification and characterization of EIDs. We explore this issue in the Western honeybee, where increasing mortality of populations in the Northern Hemisphere has caused major concern. Specifically, we investigate the importance of genetic identity of the main suspect in mortality, deformed wing virus (DWV), in driving honeybee loss. Using laboratory experiments and a systematic field survey, we demonstrate that an emerging DWV genotype (DWV-B) is more virulent than the established DWV genotype (DWV-A) and is widespread in the landscape. Furthermore, we show in a simple model that colonies infected with DWV-B collapse sooner than colonies infected with DWV-A. We also identify potential for rapid DWV evolution by revealing extensive genome-wide recombination *in vivo*. The emergence of DWV-B in naive honeybee populations, including via recombination with DWV-A, could be of significant ecological and economic importance. Our findings emphasize that knowledge of pathogen genetic identity and diversity is critical to understanding drivers of species decline.

## 1. Introduction

Emerging infectious diseases (EIDs) are a worldwide threat to biodiversity, food security and human health [1–3]. Prominent examples include lethal chytridiomycosis, a major cause of on-going amphibian species declines globally [4], and white-nose syndrome, an EID that has caused large-scale population losses of bats [5]. Recent studies have indicated a possible role for genetic diversity [6,7] and the influence of the global spread of pathogens [8] in these declines.

Bees, which provide the essential ecosystem service of pollination, are required for the production of many food crops [9]. Yet they are under pressure globally [10–18], with EIDs being implicated as a principle cause of decline [10–13,16,19,20]. Losses of the most important commercial pollinator, the Western honeybee (*Apis mellifera*), are an ongoing major concern in the Northern Hemisphere [10–12,19]. Alongside colony collapse disorder, which has so far only been observed inside the USA [12], overwinter colony loss is the principle manifestation of recent increases in honeybee decline in the Northern Hemisphere [21,22].

Deformed wing virus (DWV) and its vector, the parasitic mite *Varroa destructor* [23], have been strongly implicated as causal factors of honeybee loss. The arrival of the *V. destructor* mite precipitated a novel transmission route for viruses to enter bee haemolymph directly, resulting in the ability for DWV to generate infections very rapidly. *Varroa destructor* is linked with massively increased DWV titres and low viral genotypic diversity [24] and it is likely that emergence of RNA viruses has led to substantially decreased mite infestation thresholds for honeybee colony loss [25,26].

It is widely appreciated that DWV is a good indicator of colony decline owing to its positive temporal correlation with honeybee colony losses [27–35]. However, as with correlational relationships between other insect diseases and RNA virus infection [36], explicit tests of the relationship between defined genetic variants of DWV and honeybee host mortality have been lacking.

To understand if genetic variation in circulating honeybee pathogens could be a major determining factor of honeybee loss, we performed laboratory assays on pathogen virulence using two well-defined genotypic variants of DWV (DWV-A and DWV-B). We compared the mortality of honeybees infected with each virus genotype separately or when infected with an equal mix of both genotypes. We then asked if recombination could act a potential source of rapid evolutionary change by testing for it *in vivo* during experimental co-infection using both genotypes. Finally, we conducted a systematic field survey of Great Britain (GB) to gain an understanding of the wider prevalence of both virus genotypes in the field.

## 2. Methods

### (a) Cage experiment

Honeybee brood was collected from three colonies kept on the *V. destructor*-free island of Colonsay (Scotland). While honeybees from the island of Colonsay have never been exposed to *V. destructor*, both DWV-A and DWV-B (also known as *Varroa destructor* virus, VDV-1) can be detected there, though rarely (V. Doublet, R. J. Paxton, M. E. Natsopoulou, D. P. McMahon 2010, unpublished data), indicating that the sourced population is not entirely naive to either virus genotype. To simulate the role of viral infection through transmission by *V. destructor*, newly emerged bees were injected through the intersegmental membrane between the third and fourth abdominal segment with 1 µl of 0.5 M potassium phosphate buffer pH 8.0 (PBS) containing 10^7^ genome equivalents of DWV-A (A); DWV-B (B); DWV-A + DWV-B (5 × 10^6^ genome equivalents of each) (M); or an equivalent virus-free extract (C).

Inoculum was prepared as follows. First, batches of 10 white-eyed pupae were injected with field-derived individual bee extracts containing 10^4^ DWV-A or -B genome equivalents. Prior to propagation, field extracts were crushed in cold 0.5 M PBS (pH 8.0), using a plastic pestle, re-filtered through cotton wool, and centrifuged at 4°C for 15 min at 15 000 *g*, before extracting the supernatant, quantifying viral titres (see section Pathogen detection) and diluting in PBS to the required concentration. After 6 days, injected pupae were crushed in mesh-filtered Bio-bags (Bioreba) in cold 0.5 M PBS (pH 8.0) and purified as above. Viral titres in inocula were quantified and stored at −80°C until used in experiments. Prior to injection, the A inoculum was diluted 1.7× relative to B to equalize doses (electronic supplementary material, table S1*a*). Control inoculum was prepared from a parallel batch of uninfected white-eye pupae.

The contents of B and A inocula were analysed in detail by ultra-deep sequencing on an Illumina platform (GATC Biotech) as two separate libraries (‘M1’ and ‘M2’, respectively; electronic supplementary material, table S2). Sequenced reads were mapped to the *A. mellifera* reference genome (v. 4.5) and transcriptome (OGS v. 3.2) using Bowtie v. 2 [37]. Unmapped reads were assembled into contigs of length greater than or equal to 200 nt by the VICUNA de novo assembler [38]. We then used Bowtie v. 2 to map reads to the contigs and NCBI Blast (megablast) [39] to search for similarities between the contigs and the NCBI nucleotide collection (nt, version from 20 November 2014, downloaded from ftp://ftp.ncbi.nlm.nih.gov/blast/db). Detailed description of inoculum characterization by ultra-deep sequencing is given in the electronic supplementary material.

Experimentally injected adult bees were monitored for 24 h to confirm that mortality associated with manipulation did not exceed 10%. Bees from each source colony were mixed evenly (six per colony), held in autoclaved metal cages (18 individuals per cage) in an incubator at +30°C and dead bees were counted and removed every 24 h. Bees were fed ad libitum with 50% (w/v) sucrose solution. Each treatment consisted of four independent replicate cages. An independent subset of bees was freeze-killed in liquid N_2_ at 9 days (*n* = 5) and 13 days (*n* = 3) post-infection (p.i.) for *post hoc* virus analysis. We quantified DWV-A and -B across treatments (electronic supplementary material, table S1*b*,*c*), and confirmed that bees were negative for potential background viruses by qRT-PCR: chronic bee paralysis virus (CBPV); acute bee paralysis virus (ABPV); Israeli acute paralysis virus (IAPV); black queen cell virus (BQCV); slow bee paralysis virus (SBPV) and sac brood virus (SBV).

### (b) Pathogen detection

Total RNA of individual workers from cage experiments was obtained using the RNeasy mini kit (Qiagen) in a QIAcube robot (Qiagen) following manufacturer's instructions. Whole bees were macerated in 500 µl RLT buffer using a plastic pestle, 100 µl of which was used for RNA isolation. For the experimental inocula, 80 µl of purified supernatant was used for viral RNA isolation. In the GB field survey, methods are as previously described [20].

Total cDNA of cage experiment samples, in addition to experimental inocula, was synthetized using M-MLV Revertase (Promega) following manufacturer's instructions, using 800 ng of sample RNA. cDNA samples were diluted 1 : 10 prior to use in qRT-PCR. For absolute quantification, qRT-PCR was performed with a Bio-Rad C1000, using SYBRgreen Sensimix (Bioline) in the following programme: 5 min at 95°C, followed by 40 cycles of 10 s at 95°C, 30 s at 57°C, and 30 s at 72°C (read). RP49 was amplified for all samples as an internal reference marker. Following PCR, DNA was denatured for 1 min at 95°C and cooled to 55°C for 1 min. A melting profile was generated from 55°C to 95°C (0.5°C per second increments). We applied an upper cycle threshold (*C*_t_) of 35 for positive DWV-A and -B detection to minimize risk of false positives [40]. Absolute quantification of DWV-A and -B was calculated using duplicate DNA standard curves of purified flanking PCR products (DWV-A and -B) with efficiencies between 93% and 100% and correlation coefficients (*R*^2^) ≥ 0.988.

We employed recently developed primers [41] for amplification of the RNA-dependent RNA polymerase gene (RdRp), and confirmed DWV-A and -B primer specificity by conducting RT-PCRs from a mixed sample containing both DWV-A and -B virus, and sequencing 29 and 38 cloned PCR products for each primer pair, respectively. All sequences could be unambiguously matched to the expected virus target (electronic supplementary material, table S3). Cloning methods were as described previously [41]. A list of all primers used in PCR is given in electronic supplementary material, table S4.

### (c) Determination of recombinants

We first determined the precise sequences of the DWV-A and DWV-B genomes in our assay by aligning the Illumina sequenced reads from the inoculum datasets onto their respective reference sequences (accession numbers NC_004830.2 and NC_006494.1, respectively) using Bowtie v. 2 [37] (electronic supplementary material, figure S1*a*) and generating consensus sequences from the aligned sequencing reads. The DWV-A consensus differed by 200 bp (198 mismatches, two insertions; 98.0% sequence similarity) and the DWV-B consensus sequence by 76 bp (75 mismatches, one insertion, 99.3% sequence similarity) from their respective reference sequences. The sequence similarity of our genome consensus for DWV-A to our genome consensus for DWV-B was 84.2% (1580 mismatches, 28 insertions and deletions; electronic supplementary material, figure S2*a*) and each genotype varied by less than 0.1% across its genome (electronic supplementary material, figure S1*a*), indicating that the two viral inocula did not form a single, interconnected mutant cloud or quasi-species.

Illumina sequencing data were generated from a third library (‘M3’) of five pooled 9d p.i. M-treated honeybees (23 546 472 reads in total) and were processed as follows. First, all sequencing read pairs with overlapping ends (2 602 106 reads, 11.1%) were discarded from the analysis. The remaining reads pairs were mapped on both consensus sequences. We assumed that a single read (101 bp) originated from DWV-A rather than DWV-B if it matched with at most one mismatch to DWV-A, and with at least six further mismatches to DWV-B. Conversely, we assumed that a single read originated from DWV-B rather than DWV-A if it matched with at most one mismatch to DWV-B, and with at least six further mismatches to DWV-A. We then defined recombinant read pairs as ‘discordant’ if they met all four of our criteria: (i) one read of a pair originated from DWV-A and the other from DWV-B; (ii) the mapping positions of the two read ends did not overlap; (iii) the read with the lower mapping position was mapped on the plus strand of the virus genome while the other read with the higher mapping position was mapped on the minus strand; and (iv) the distance between the 5′ ends of the mapped read ends (i.e. the fragment length) was at most 500 bp.

### (d) Great Britain survey of deformed wing virus-A and -B

Honeybee foragers from 25 sites across GB (electronic supplementary material, figure S3) were previously screened for DWV-related viruses [41], wherein the RdRp region was amplified by qRT-PCR to assess the prevalence and individual loads of viruses belonging to the DWV complex (including DWV-A and -B). We re-examined the data by treating DWV-A and -B results separately.

### (e) Statistical analyses

**Figure 1.**
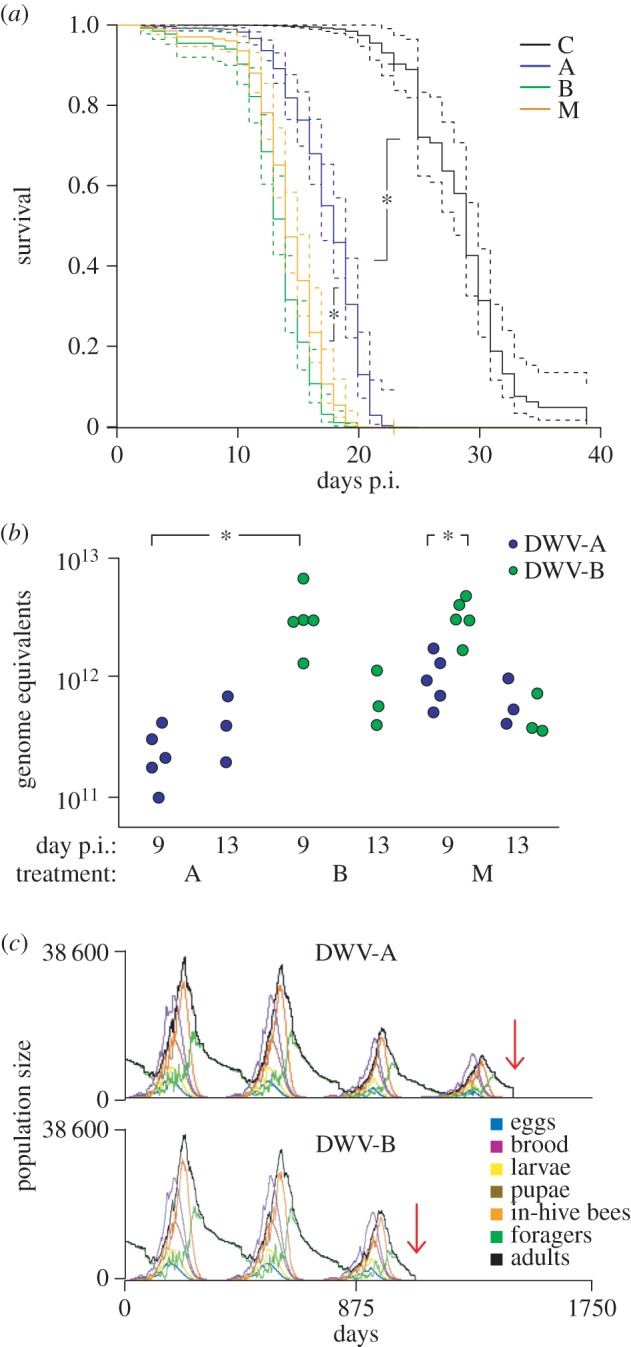
Test of DWV virulence under controlled conditions. (*a*) Fitted Cox proportional hazard survival curves (solid coloured lines) in days post-infection (p.i.) following exposure by injection of *V. destructor*-free, newly emerged adults. C = control (black); A = DWV-A (blue); B = DWV-B (green); M = mix (orange) and 95% CIs for each fitted curve (dashed coloured lines). Star/lines show significant differences between treatments (*p* < 0.05) based on *post hoc* pairwise comparisons of the final model in table 1. Median survival of control bees was 29 days, for DWV-A injected bees it was 18 days, and for DWV-B injected bees it was 13.5 days. (*b*) DWV-A and -B titres in A, B and M treatments from bees extracted at 9 and 13 days p.i. (*c*) Population dynamics over time of colonies infected with DWV-A or -B. Models were run in BEEHAVE [45] with Mite-model parameters adjusted to reflect the relative individual mortality of adults and pupae infected with either DWV-A or -B. Individual daily mortality rates were derived from laboratory experiment survival data (panel (*a*); described in Methods). Colony collapse events are indicated by a vertical red arrow.

All analyses were performed in R v. 3.1.3 [42]. Survivorship of experimentally inoculated bees was analysed, using Cox proportional hazard models (R packages ‘coxme’ and ‘coxph’) [43,44]. Models contained cage-replicate and treatment as random and fixed effects, respectively (‘coxme’; table 1), except where survival curves were depicted graphically in that case ‘cage’ as a random term could not be incorporated into curve fitting (‘Survfit’ function of a ‘coxph’ object; figure 1; electronic supplementary material, figure S4). The R package ‘multcomp’ [46] was used for comparing significant differences between treatment means, using Bonferroni correction to account for multiple testing. Moran's *I* indices were estimated in ‘ape’ [47]. All raw data files and code used in analyses are available in Dryad [48]. Sequenced Illumina reads from the DWV-A and -B inocula (libraries M2 and M1 respectively) and mixed-infection library (M3) are available from the Sequence Read Archive (BioProject PRJNA325785) and sequenced cloned PCR products are available from GenBank (accession nos. KX265618–KX265684).

**Table 1.** Final Cox proportional hazard model of cage mortality following experimental inoculation. (C, control; A, DWV-A; B, DWV-B; M, mixed DWV-A and -B. s.e., standard error; s.d., standard deviation.)

	coefficients			model testing		
parameters	*β*	s.e. (*β*)	Exp. (*β*)^a^	*χ*^2^ (LRT)	d.f.	*p*-value
fixed variable						
treatment				50.706	3	<0.00001*
C	0	—	1			
A	4.643	0.619	103.865			
B	6.480	0.643	651.826			
M	6.093	0.638	442.705			
random variable	s.d.	variance				
cage	0.403	0.162				

^a^Equivalent to the hazard ratio, the instantaneous risk of death for bees in each treatment compared with the baseline treatment level (in this case C). Higher levels of β indicate higher risk of death.

The impact of DWV-A and -B on colony survival was explored using the BEEHAVE-Model (BEEHAVE-Model version 2014-03-04, www.beehave-model.net) [45]. BEEHAVE simulates realistic honeybee colony growth and foraging dynamics and can be used to explore how honeybee colonies may be impacted by stressors manipulated either singly or in combination. To simulate the effect of DWV-A and -B on colony performance, the Mite-module within the BEEHAVE model was employed. Two scenarios were compared: (i) default settings with *Varroa*/DWV (i.e. DWV-A) infection as described in reference [48] (10 virus-free mites and 10 virus-carrying mites introduced on day 0); and (ii) As setting (i) but with simulated infection by DWV-B, corresponding to a 0.4% increase in daily mortality rate of adult bees compared with DWV-A. Daily mortality rates of bees were derived from our experimental data at the day of 50% survivorship (1 – [^median survival^ √0.5] = daily mortality rate). Model mortality rates were calculated by calibrating the daily cage experiment mortality rate to the default values in the BEEHAVE model (0.004 and 0.012 for background and DWV-A mortality, respectively). The relative mortality rate of DWV-B used in scenario (ii) was then extrapolated from these values (i.e. 0.016). We applied these values to two variables controlling adult and pupal mortality, respectively, but a conservative model was also conducted where only the adult mortality rate was altered. Both scenarios were run until the death of the colony (i.e. a colony was presumed dead when there were less than 4000 bees at the end of a calendar year). A detailed description of the adjusted model parameters is given in the electronic supplementary material, table S5.

## 3. Results

### (a) Cage experiment

Characterization of experimental inocula by ultra-deep sequencing revealed very low variability within (*ca* 0.04%) but high variability between (*ca* 16%) DWV-A and -B inocula. We found no evidence of co-occurring honeybee-associated organisms, inclusive of another recently characterized DWV genotype (DWV-C; electronic supplementary material, table S2 and figures S1, S2)

We experimentally exposed naive adult workers to inocula containing field-derived DWV-A and -B in the absence of its biological vector, *V. destructor*, to investigate the importance of DWV genetic variation for honeybee host virulence (figure 1*a*). Median survival in days for treatments C, A, B and M was as follows (95% CIs): 29 (28–30), 18 (18–19), 13.5 (13–14) and 14 (13–14), corresponding to a daily mortality rate of 0.024, 0.038, 0.050 and 0.048, respectively. Survival was significantly reduced by all virus treatments compared with the control (Tukey *post hoc* comparison of model means (Bonferroni corrected): B versus C: *z* = −10.08, *p* < 0.0001; A versus C: −7.503, *p* < 0.0001; M versus C: *z* = −9.552, *p* < 0.0001). B was significantly more virulent than A (*z* = −5.317, *p* < 0.0001). Furthermore, M was significantly more virulent than A (*z* = −4.276, *p* < 0.0005), but not B (*z* = 1.167, *p* = 1.00). The final model output is given in table 1.

A *post hoc* qRT-PCR screen of a subsample of bees collected 9d and 13d p.i. showed that virus-injected bees each contained more than 10^11^ DWV-A or -B genome equivalents in the A and B-injected treatments, respectively, and that C-injected bees were free of both viruses (electronic supplementary material, table S1). Additionally, DWV-B titres in B-injected bees were higher than DWV-A titres in A-injected bees at 9d p.i. but not 13d p.i. (Wilcoxon signed-rank test *W* = 0, *p* < 0.001 and *W* = 2, *p* = 0.4, respectively), possibly because of mortality of heavily infected bees from treatment B between 9d and 13d p.i. Likewise, DWV-B titres were higher than DWV-A titres in M-injected bees at 9d p.i. but not 13d p.i. (Wilcoxon signed-rank test *W* = 0, *p* < 0.001 and *W* = 2, *p* = 0.4, respectively; figure 1*b*). We also found that some B-injected bees sampled at 9d p.i. contained very low levels of DWV-A but that bees sampled at 13d p.i. were free of the co-occurring virus (electronic supplementary material, table S1).

To confirm that differential virulence was owing to inherent differences between DWV-A and -B and not owing to the different composition of the starting inocula, we reanalysed mortality across the treatments starting from day 13, where sampled bees did not contain co-occurring viruses. These yielded the same results as above between all treatment comparisons (electronic supplementary material, figure S4 and table S6): B versus C: *z* = −9.147, *p* < 0.0001; A versus C: *z* = −7.067, *p* < 0.0001; M versus C: *z* = −8.685, *p* < 0.0001; M versus A: *z* = −3.008, *p* = 0.016; M versus B: *z* = 0.900, *p* = 1.0; A versus B: *z* = −3.832, *p* = 0.0008.

To understand whether the differences in DWV-A and -B virulence detected in the laboratory were also meaningful at the colony-level, we modelled whole colony population dynamics using individual bee mortality rates extrapolated from our laboratory survival data (see Methods). We found that, when infected with DWV-A, colonies survived four complete annual cycles, whereas colonies that were infected with DWV-B survived only three complete annual cycles (figure 1*c*). Regardless of whether model scenario (i) or (ii) was used (electronic supplementary material, figure S5), colony collapse occurred during the fourth and fifth winter for DWV-B and -A infected colonies, respectively.

### (b) Recombination

In total, we detected 19 984 discordant read pairs, which were used to infer recombinants (electronic supplementary material, figure S2*b*); both read pairs of 13 513 751 sequences matched uniquely to either DWV-A or -B. This re-confirmed that experimental honeybees contained high titres of DWV-A and -B to the exclusion of other common honeybee viruses or unintended DWV genotypes (DWV-C). Significantly, our analyses of discordant read-pairs revealed extensive evidence of recombination across the genome (electronic supplementary material, figure S2).

### (c) Field prevalence: country-wide survey of deformed wing virus -A and deformed wing virus-B

**Figure 2.**
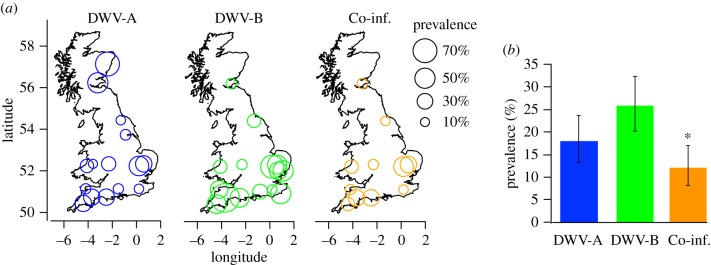
Prevalence of DWV-A and -B in honeybees collected during a structured sampling survey of GB (%). (*a*) Estimated prevalence of DWV-A (blue), DWV-B (green) and putative co-infections (orange) are indicated by variable circle areas at sampling locations. Sample sites with zero prevalence not indicated (see the electronic supplementary material; figure S3 for sample site information). (*b*) True prevalence estimates for each virus, including 95% CIs (%), with significant non-independence (χ^2^ test) of the two virus genotypes indicated by an asterisk over the co-infection (orange) column.

We examined the data from a recent field survey of honeybee foragers at 25 sites across GB [41] to understand the prevalence and therefore the wider potential impact of DWV-B across honeybee populations. We found that DWV-B was more prevalent than DWV-A, but this difference was marginally non-significant in a test of proportions (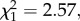 *p* = 0.055; figure 2*a*). The spatial distribution of viruses also differed (figure 2). DWV-B was largely restricted to southern England, and was significantly clustered (Moran's *I* = 0.12, *p* = 0.005), whereas DWV-A was more uniformly distributed across GB (Moran's *I* = −0.087, *p* = 0.432; figure 2*b*). Finally, the number of co-infected individuals was higher than expected by chance (11.45% observed versus 4.41% expected, *χ*^2^ = 37.92, *p* < 0.0001), indicating non-independence of DWV-A and -B infections among honeybee foragers.

## 4. Discussion

Our findings reveal that: (i) a recently described genotypic variant of DWV [49], described here as DWV-B following [50], is more virulent than the established DWV-A genotype in a controlled laboratory assay; (ii) the two genotypes readily recombine *in vivo*; and (iii) DWV-B is geographically widespread. Our findings demonstrate that DWV is composed of at least two divergent genotypes that differ significantly in biology, and that DWV-B, either singly or as a recombinant, may represent a particular threat to naive honeybee populations.

The ongoing biodiversity crisis is exacerbated by interactions among a multitude of stressors, including pathogens, pesticides, habitat degradation, overexploitation, climate change and exotics introduced through commercial trade [16,51]. In honeybees, the arrival of a novel disease vector in the form of *V. destructor* created an opportunity for dramatically increased pathogen transmission, which may have triggered a shift towards increased virulence in viruses such as DWV that were already present in honeybee populations, but had previously persisted as low-level asymptomatic infections. However, whether emerging pathogen diversity itself could be a major driving force of honeybee population decline, as may be the case in amphibians [6,7,52], cannot yet be determined.

The status of DWV-B outside of Europe is not well understood. A recent study implicated DWV-B alongside DWV-A and *V. destructor* infestation as a causal factor in colony decline in the southern USA [34], although DWV genotypes were identified indirectly in this study (by probe hybridization and not by sequencing). Another study did not detect significant levels of DWV-B in North America [53]. These findings highlight the considerable lack of understanding concerning the global distribution of genotypic variants of DWV. A recent analysis found the global pandemic of DWV to be mediated by European honeybee populations [54], and that North America acts as an important hub for DWV-A into the New World. This demonstrates that Europe may be an important source of and/or route for the global spread of emerging and re-emerging DWV strains. Unfortunately, detailed knowledge of the global phylogeography of genotypes such as DWV-B is currently lacking. For example, it is possible that the spread of DWV-B to the USA occurred recently and/or is currently ongoing. However, this remains speculative until a systematic survey of viral population variation is conducted. Such information is needed to improve knowledge of the origins, levels of endemism and current prevalence of different DWV genotypes.

In addition to field studies, laboratory experiments are required to assess comparative virulence across a broader range of representative DWV and host genotypes. For example, a novel DWV-C genotype has also recently been reported in the UK and Hawaii [50] but detailed knowledge concerning its origins, global prevalence and impact both in the field and under controlled laboratory conditions are needed. Because our study reports virulence from a single population, further research is also required to test the extent to which elevated DWV-B virulence can be extrapolated to other host honeybee genotypes. The combination of such data can then be used to assess the potential risk of emergence of specific viral genotypes to honeybee populations across temperate zones.

While DWV-A and -B are justifiably considered to belong to the same complex due to nucleotide sequence and proteolytic cleavage site similarity [55], our data demonstrate that considering them as synonymous in terms of their underlying biology could be misleading. DWV-A and -B have been described as belonging to a single quasi-species [55], but given the genetic distance separating the two genotypes (approx. 16% at the nucleotide level), in addition to marked geographical and phenotypic differences that we show here to result in a significant difference in host fitness, we argue that it is unhelpful to consider DWV-A and -B as epidemiologically or evolutionarily equivalent. Indeed, these genotypes are the result of evolutionary processes that have taken place over medium-term timescales [50,54]. Use of the term ‘genotype’ to describe DWV-A and -B is consistent with virological nomenclature, where a ‘genotype of a virus can be viewed as a set of related genomes that have found a high fitness domain and acquired epidemiological relevance associated with replicative or non-replicative traits' [56].

In a comparative test of mortality in naive bees, our data show that DWV-B is more virulent than DWV-A and that this may be attributable to a superior rate of replication in honeybees. The difference in virulence we detected translates into a reduction in median lifespan of 38% for DWV-A injected bees versus 53.5% for DWV-B, against control-injected bees. Such a decrease in the median lifespan of the workforce could have a significant impact on the colony as a whole. We tested this by modelling colony populations in the presence of either DWV-A or -B, finding that colonies collapse 1 year earlier when infected with DWV-B when compared with DWV-A. This is in agreement with findings from RNA viruses and other pathogens in other systems, where significant differences in virulence among closely related genotypes are commonplace for diseases of both humans [57–59] and wildlife [60,61], with major repercussions for transmission dynamics and epidemic potential. While this possibility has been widely discussed for bee viruses [32,62–66], it has not been explicitly tested until now.

Recombination may act as a powerful generator of chimeric DWV genotypes [67–69], which may lead to significant genotype mixing. For instance, our field data demonstrate that forager honeybees co-infected with both DWV-A and -B occur more commonly than expected by chance, though the extent to which this reflects recombination is unclear. We also show in an experimental setting that DWV-A and -B readily recombine at sites across the genome when the two genotypes are present at high levels during co-infection. Together, these point to potential for extensive generation of recombinant genotypes via genome-wide recombination mechanisms, although the extent of recombination actually taking place in the field still remains to be determined. Such processes could in theory have important knock-on effects for long-term virulence evolution and host adaptation [70]. Indeed, naturally occurring recombinants composed of DWV-A and -B have recently been reported [67,68], and the discovery of chimeric DWV-A/B viruses has been linked to higher virulence in honeybee pupae [69]. For example, a recent report of superinfection exclusion of DWV-A by DWV-B [71] could also be owing to a novel A/B recombinant.

It has been hypothesized that DWV-B derived capsid peptides could facilitate horizontal transmission via *V. destructor* and that DWV-A derived non-structural proteins (e.g. internal ribosome entry site) might permit higher (host-specific) replication in honeybee cells [67]. However, specific associations between DWV-B and *V. destructor*, or between DWV-A and *A. mellifera* have not been empirically tested. Additionally, aside from the *V. destructor* sample from which it was isolated, we see no *a priori* reason to suspect that the DWV-B genotype might be more or less adapted to either *V. destructor* or *A. mellifera*, when compared with DWV-A. Rather, our findings indicate that DWV-B replication in *A. mellifera* may in fact be superior to that of DWV-A. Regardless of any uncertainty over the putative adaptive origins of DWV-A and -B, the potential for novel strains with significantly altered virulence dynamics to emerge via recombination should be acknowledged, and incorporated into future honeybee pathogen detection and mitigation strategies.

## 5. Conclusion

DWV-B is widespread in the landscape and it is more virulent than the original DWV-A genotype in the laboratory. Explaining how *V. destructor* influences epidemiology, both as a biological vector and putative second host, is central to improving our understanding of the continuing impact of both DWV-A and -B [72]. In this regard, we argue that the explicit inclusion of honeybee—*V. destructor*— virus interactions into related research questions such as intracolony dynamics [23,73] and immunity (particularly in relation to pesticide use) [74] is important. The continuing decline of wild pollinators such as bumblebees [13–15], alongside recent evidence of widespread and on-going spillover of *V. destructor* vectored RNA viruses between managed and wild bees [20,41], also demonstrates the wider potential for disease emergence in other bee pollinators. Our findings emphasize the importance of understanding the wider extent of pathogen genetic diversity when investigating causes of species decline, and highlight the need for a coordinated effort to tackle directly the issue of mite-vectored viruses in honeybees.

## Supplementary Material

Supporting Information

## Data accessibility

All raw data files and code used in analyses are available in Dryad (doi:10.5061/dryad.cq7t1). Sequenced Illumina reads from libraries M1-M3 are available from NCBI (BioProject PRJNA325785) and sequenced cloned PCR products are available from Genbank (KX265618–KX265684).
