# Supplementary material for: Elevated virulence of an emerging viral genotype as a driver of honeybee loss
Source: Proc Biol Sci. 2016 Jun 29;283(1833):20160811. doi: 10.1098/rspb.2016.0811 (PMC4936039; doi:10.1098/rspb.2016.0811)
Supplement: Supporting Information [file rspb20160811supp1.pdf]

Supporting Information

Elevated virulence of an emerging viral genotype as a driver of honeybee loss

Dino P. McMahon, Myrsini E. Natsopoulou, Vincent Doublet, Matthias Fürst, Silvio Weging, Mark J. F. Brown, Andreas Gogol-Döring, Robert J. Paxton

Contents

- Supplementary Methods
- Supplementary Figures
- Supplementary Tables

Supplementary Methods: Characterization of experimental inocula via ultra-deep sequencing.

After mapping reads to contigs using Bowtie2 [1], we used NCBI Blast (megablast) [2] to search for similarities between the contigs and the NCBI nucleotide collection (nt, version from November 20th 2014, downloaded from ftp://ftp.ncbi.nlm.nih.gov/blast/db). Most contigs matched nucleotide sequences originating from Apinae spp., so we assigned the corresponding sequencing reads to *A. mellifera*. Other contigs represented the bee viruses DWV-A or -B. Reads that mapped equally well to both viruses were counted as either DWV-A or -B in the same proportion as the reads that could be uniquely assigned to either DWV-A or -B; this is an unbiased approach because, though sequence similarity of DWV-A and -B varies across the length of the genome (Fig. S1), reads that could be mapped to both variants were spread evenly across the consensus genome in proportion to sequence similarity of the DWV genotypes A and B (Fig. S1). Some contigs were identified as the bacteriophage phiX, which is widely used for Illumina sequencing control libraries, so we assume that these reads are technical artefacts. A manual inspection of the Blast results revealed that the remaining contigs contained either very unspecific sequences or were assigned to database entries clearly unrelated to *A. mellifera*. All reads either corresponding to these unspecific or unrelated contigs, or to contigs which could not be found in the NCBI nucleotide collection, together with all reads which could not be mapped to any contig, were further processed as follows. After filtering out low-complexity sequences with PRINSEQ [3] (threshold 15 for 'dust' and 85 for 'entropy'), reads were mapped with Bowtie2 to the NCBI virus database [4]. No bee-related virus other than the viruses mentioned above could be found this way. Then

we mapped all remaining reads one by one to the NCBI nucleotide collection using blastn [5]. In this way, we sorted these reads into the classes described before, but we could not identify any further bee-related or bee-associated organism. At the end, only 47,587 (0.23%) reads from the B inoculum and 12,434 (0.064%) reads from the A inoculum remained unassigned. Both inoculum libraries were sequenced on the same multiplexed Illumina flow cell lane, so a small fraction of sequencing reads is expected to be accidently assigned to the wrong data set, e.g. because of errors during barcode sequencing or due to chimeric reads. This could explain the very low occurrence of DWV-B in the A inoculum data set and also a part of the few DWV-A reads in the B inoculum data set. We also compared the average variability of each inoculum by calculating the proportion of nucleotide mismatches, insertions and deletions between the reads and the respective consensus genome sequences. Average mutation frequencies were calculated inside of non-overlapping 100 base pair windows (Fig. S1). Sequencing errors were avoided by conducting a restricted analysis using only identical overlapping paired-end read portions. The detected levels of variability were very low and similar for both the A and B inocula (~0.04%).

Finally, we analyzed reads from a third library prepared from RNA extracted from cage experiment-derived honeybees (N=5 pooled 9d p.i. M-treated honeybees, in total 23,546,472 reads) in order to search for the presence of a recently characterized genotype of DWV: DWV-C [6]. Reads were concordantly mapped using Bowtie2 to a single index containing the genomes of DWV-A/Kakugo virus (accession numbers NC_004830.2 and NC_005876.1 respectively), DWV-B (NC_006494.1) and DWV-C (gi|873406561|emb|CEND01000001.1) allowing for multiple hits. All reads that mapped to DWV-C also mapped to one of the other reference genomes with the same

or better alignment score, so we concluded that DWV-C was not present in the experimental inocula.

SI References

1. Langmead, B. & Salzberg, S.L. Fast gapped-read alignment with Bowtie 2. *Nat Methods* **9**, 357-359 (2012)
2. McGinnis, S. & Madden T.L. BLAST: at the core of a powerful and diverse set of sequence analysis tools. *Nucleic Acids Res* **32**, W20-25 (2004).
3. Schmieder, R. & Edwards, R. Quality control and preprocessing of metagenomic datasets. *Bioinformatics* **27**, 863-864 (2011).
4. Bao, Y. *et al.* National center for biotechnology information viral genomes project. *J Virol* **78**, 7291-7298 (2004).
5. Altschul, S. F., Gish, M., Miller, W., Myers, E. W. & Lipman, D. J. Basic local alignment search tool. *J Mol Biol* **215**, 403-410 (1990).
6. Mordecai, G. J., Wilfert, L., Martin, S. J., Jones, I. M., Schroeder, D. C. Diversity in a honey bee pathogen: first report of a third master variant of Deformed wing virus quasispecies. *ISME J.* Early view (2015).

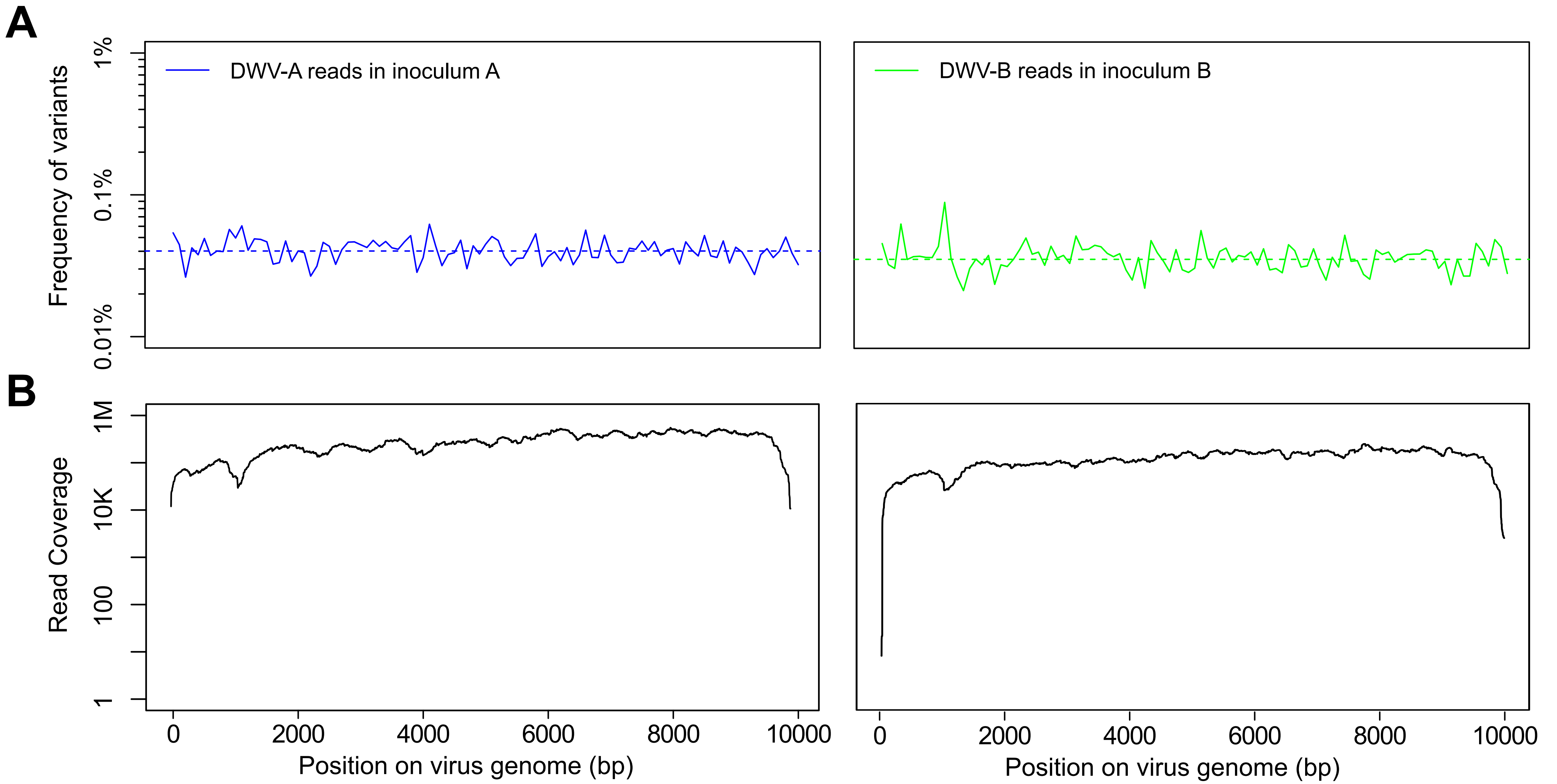

Fig. S1. Genetic variability and read coverage of the experimental inocula. (**A**) Average genome-wide variability of DWV-A in the A inoculum, and DWV-B in the B inoculum respectively. These represent nucleotide mismatches, insertions and deletions between DWV-A or -B reads when matched against their respective consensus genome sequences. Mutational variation around each isolate (DWV-A or -B) derived from field-infected bees was <1% whereas sequence divergence between isolates was >15% (Fig. S2), indicating that the two viral genotypes do not form an interconnected mutant cloud. (**B**) Genome-wide coverage of DWV-A and DWV-B reads in A and B inocula, respectively.

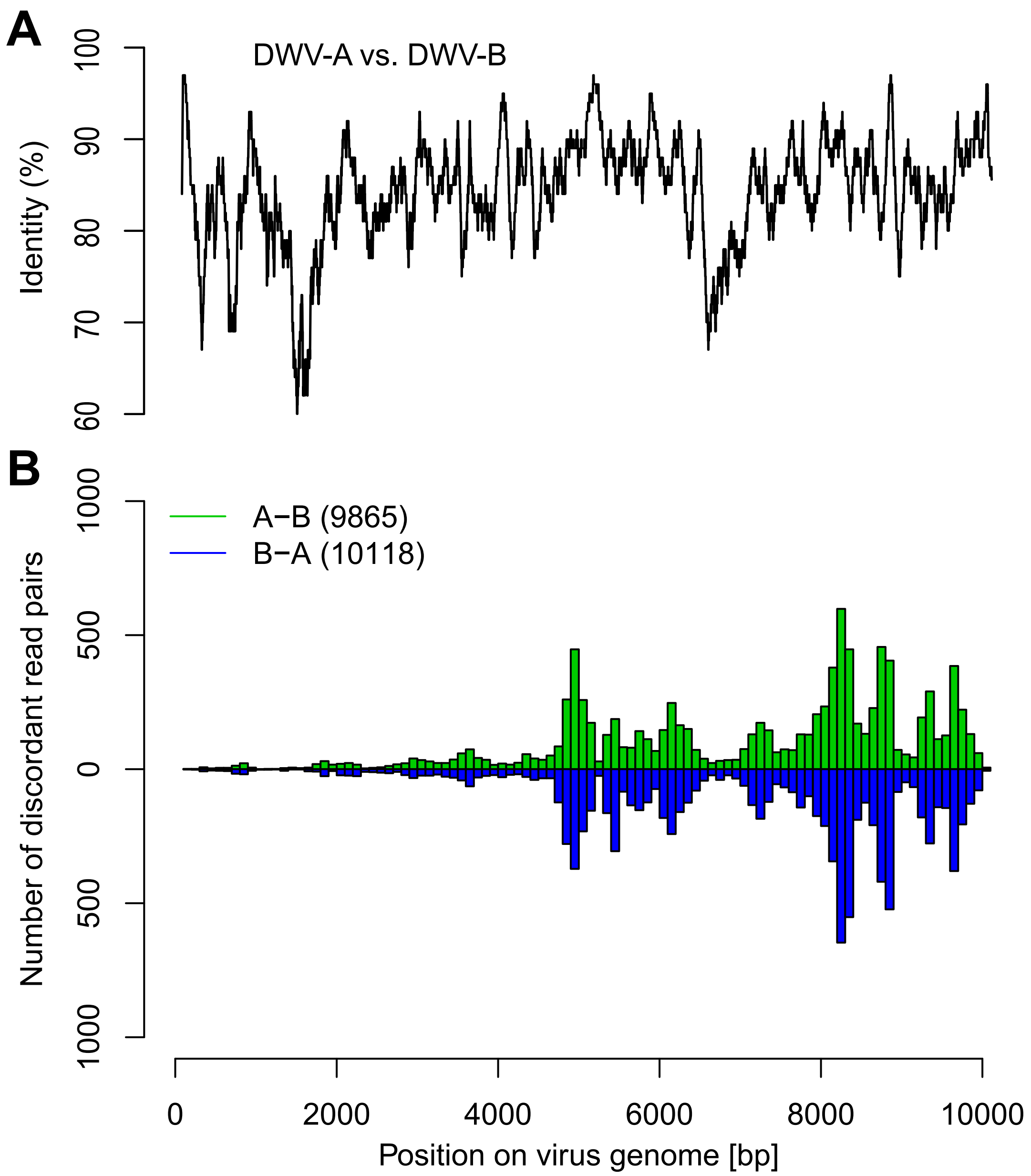

Fig. S2. Genome-wide sequence similarity and extent of recombination between DWV-A and -B. **(A)** Genome-wide sequence similarity of DWV-A and -B (derived from consensus sequences of experimental inocula) in a 100bp sliding window. **(B)** Genome-wide rate of recombination between DWV-A and -B genomes as inferred by number of DWV-A and -B discordant read-pairs from pooled M-treated individuals 9d p.i. (N=5). The length of each bar in the histogram corresponds to the number of discordant read pairs whose centers fall into a 100bp window of the virus genome. The green bars give the number of recombinants with a DWV-A to -B fusion; the blue bars give the number of recombinants with a DWV-B to -A fusion.

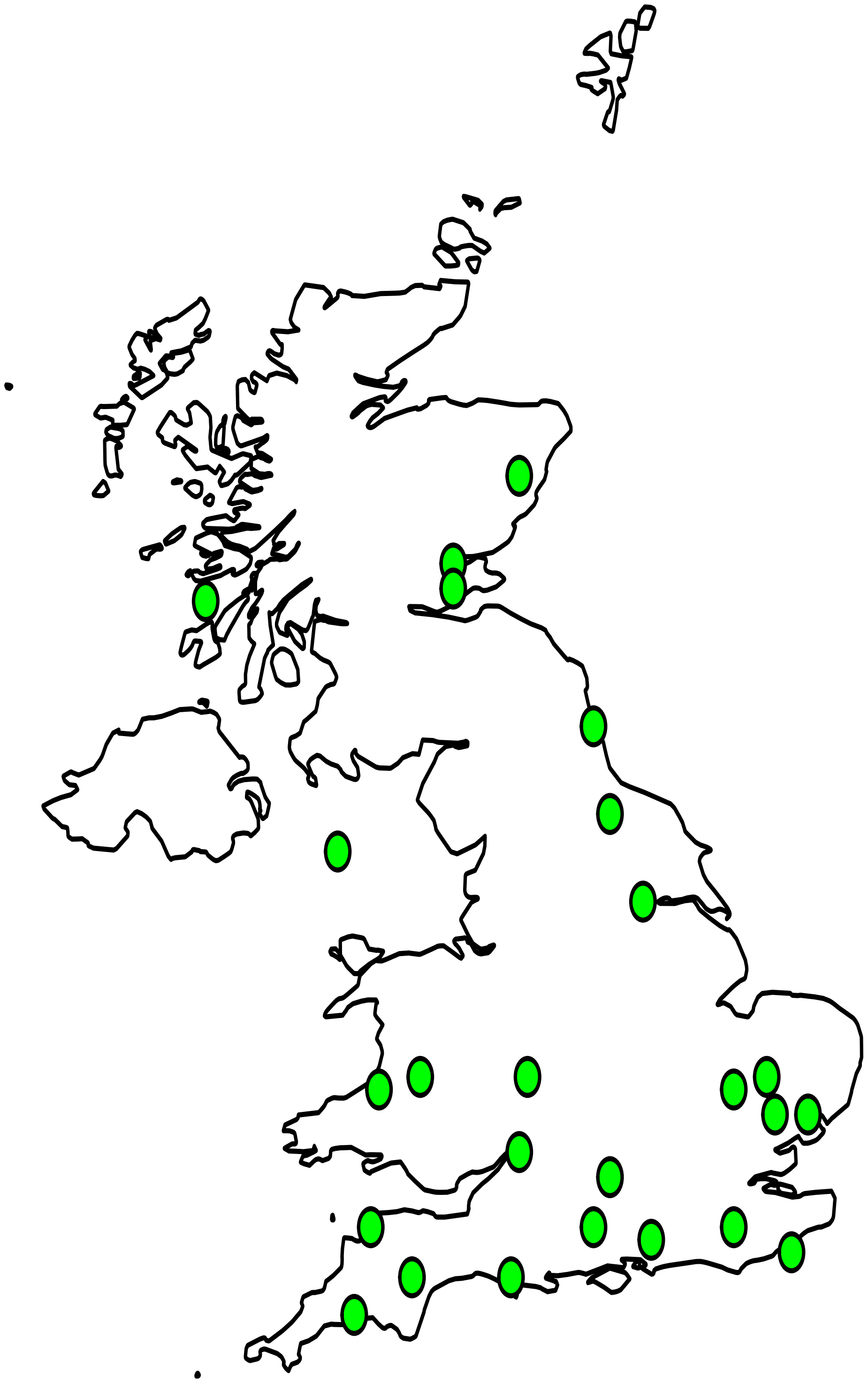

Fig. S3. Location of sampling sites used in the GB survey [20,41].

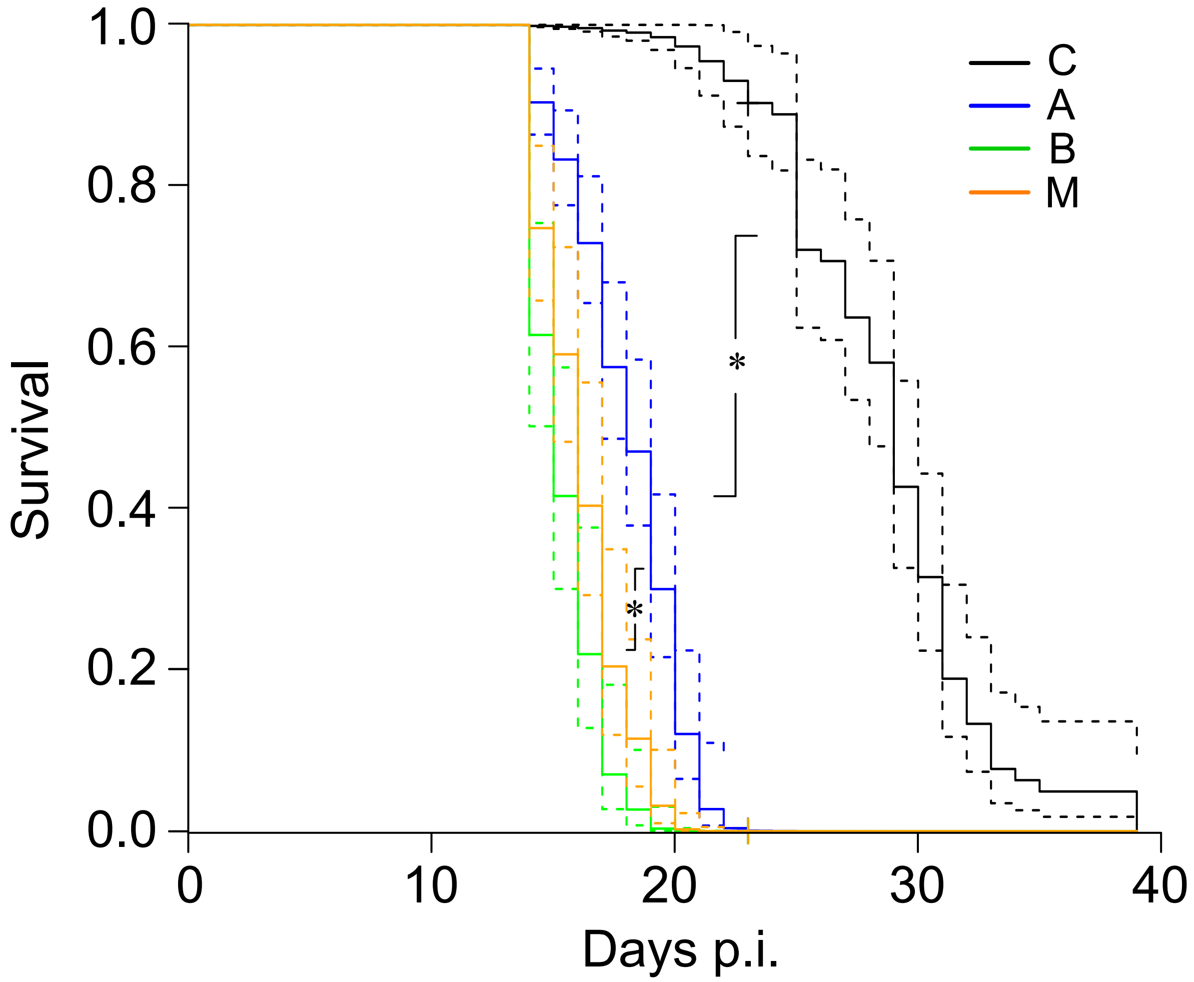

Fig. S4. Fitted Cox proportional hazard survival curves (solid coloured lines) of bees from 13 days p.i. onwards. C=Control (black); A=DWV-A (blue); B=DWV-B (green); M=Mix (orange) and 95% CIs for each fitted curve (dashed coloured lines). Star/lines show significant differences between treatments (P<0.05) based on post-hoc pairwise comparisons of the final model in Table S6.

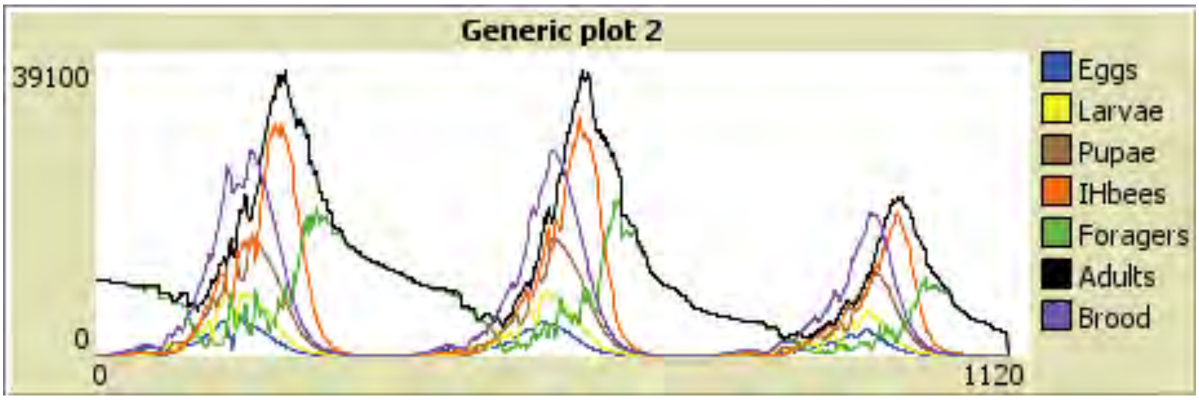

Fig. S5. Population dynamics over time of colonies infected with DWV-B, with modifications only to the relative individual mortality of adult bees: the individual daily mortality rate of pupae was kept at the default setting (Table S5). The model was run in BEEHAVE [48]

Table S1. (**A**) Virus loads (as genome equivalents) of A and B inocula used to calculate infectious dose. Virus loads (qRT-PCR) from cage experiment individuals sampled at 9d p.i. (**B**) and 13d p.i. (**B**). Ct values transformed into virus genome equivalents per honeybee are shown.

A.

INOCULA

DWV-B		DWV-A	
Ct value	Q / genomes / ul	Ct value	Q / genomes / ul
15.1	6.97 x 10^8	13.46	1.18 x 10^9 §

§ The A inoculum was diluted x1.69 relative to the B inoculum to equalize doses. This is consistent with the proportion of DWV-A reads compared to DWV-B reads in the illumina-sequenced A and B inocula, respectively (Table S2.)

B.

9 DAYS P.I.

Treatment	Sample	DWV-B		DWV-A		RP49
		Ct value	Absolute quantity	Ct value	Absolute quantity	Ct value
C	C1	na	na	***37.69***		24.99
	C2	na	na	***37.30***		24.43
	C3	na	na	na	na	24.05
	C4	na	na	***37.62***		24.27
	C5	na	na	***37.74***		24.81
A	D1	***36.05***		11.96	176569565921.80	25.09
	D2	na	na	11.10	414617806383.81	24.37
	D3	***36.39***		11.14	212221814165.47	24.03
	D4	35.16	18916.76	11.56	302548758497.88	24.33
	D5	na	na	12.08	99653859170.76	24.68
B	V1	10.08	2920480607485.87	34.50	120113.93	26.43
	V2	9.17	2901401640057.17	***35.51***		26.04
	V3	9.35	2820652080550.68	31.60	496756.50	25.37
	V4	9.88	1280784021269.72	33.81	73550.91	25.38
	V5	9.27	6424227863871.44	32.75	498041.52	25.43
M	M1	10.10	1645366097189.03	10.81	504504218346.07	25.82
	M2	9.95	4607122304117.58	10.38	1699703416392.77	25.48
	M3	10.40	2949987704299.03	11.51	692417826596.36	26.27
	M4	10.16	2897697469151.58	10.81	925610286072.58	25.42
	M5	9.80	3892980228102.32	10.41	1285922177392.84	25.55

Bold italic Ct values > acceptance threshold (Ct = 35). na = not detected

Table S1. Cont.

C.

13 DAYS P.I.

Treatment	Sample	DWV-B		DWV-A		RP49
		Ct value	Absolute quantity	Ct value	Absolute quantity	Ct value
C	C6	na	na	na	na	24.48
	C7	na	na	na	na	24.32
	C8	na	na	na	na	23.99
A	D6	na	na	10.26	195511930427.82	25.37
	D7	na	na	9.96	683595893151.01	25.19
	D8	na	na	9.87	391146603892.63	24.48
B	V6	10.86	396656612110.95	na	na	25.30
	V7	10.99	566967760401.87	na	na	25.49
	V8	11.01	1116755448574.56	na	na	25.75
M	M6	12.04	375004409986.09	10.85	406282317906.32	25.54
	M7	11.58	722585275438.26	10.09	962219268280.72	24.90
	M8	12.37	355406511228.20	10.68	533357594158.83	25.50

na = not detected

Table S2. Origin of sequenced reads in the DWV-A and -B inocula (see Supplementary Methods for detailed description of methods).

Origin	B inoculum reads (M2)	A inoculum reads (M1)
Apis	15,277,458 *(74.5%)*	8,545,289 *(44.2%)*
Viruses		
DWV-B	4,547,491 *(22.2%)*	623 *(0.0032%)*
DWV-A	6,157 *(0.03%)*	10,334,080 *(53.5%)*
Overhead		
phiX	26,355 *(0.13%)*	28,010 *(0.14%)*
sequencing adapters	3,555 *(0.017%)*	4,327 *(0.022%)*
low complexity	602,276 *(2.9%)*	405,663 *(2.1%)*
Unknown	47,587 *(0.23%)*	12,434 *(0.064%)*
Total	20,510,879	19,330,426

Table S3. Sequence alignment of cloned viral RdRp gene PCR products, amplified using specific (qRT-PCR) DWV-A and -B primers from a mixed-template. Sequences are compared against DWV-A (GenBank Accession No. NC004830) and DWV-B (GenBank Accession No. NC006494) reference genes.

DWV_B_NC_006494	CTTCTGGTAA	GCGATGGTTG	TTTGATATTG	AATTACAAGA	TTCAGGATGT	TATCTTTTGA
DWV_B_clone1	CTTCTGGTAA	GCGATGGTTG	TTTGATATTG	AATTACAAGA	TTCAGGATGT	TATCTTTTGA
DWV_B_clone2	CTTCTGGTAA	GCGATGGTTG	TTTGATATTG	AATTACAAGA	TTCAGGATGT	TATCTTTTGA
DWV_B_clone3	CTTCTGGTAA	GCGATGGTTG	TTTGATATTG	AATTACAAGA	TTCAGGATGT	TATCTTTTGA
DWV_B_clone4	CTTCTGGTAA	GCGATGGTTG	TTTGATATTG	AATTACAAGA	TTCAGGATGT	TATCTTTTGA
DWV_B_clone5	CTTCTGGTAA	GCGATGGTTG	TTTGATATTG	AATTACAAGA	TTCAGGATGT	TATCTTTTGA
DWV_B_clone6	CTTCTGGTAA	GCGATGGTTG	TTTGATATTG	AATTACAAGA	TTCAGGATGT	TATCTTTTGA
DWV_B_clone7	CTTCTGGTAA	GCGATGGTTG	TTTGATATTG	AATTACAAGA	TTCAGGATGT	TATCTTTTGA
DWV_B_clone8	CTTCTGGTAA	GCGATGGTTG	TTTGATATTG	AATTACAAGA	TTCAGGATGT	TATCTTTTGA
DWV_B_clone9	CTTCTGGTAA	GCGATGGTTG	TTTGATATTG	AATTACAAGA	TTCAGGATGT	TATCTTTTGA
DWV_B_clone10	CTTCTGGTAA	GCGATGGTTG	TTTGATATTG	AATTACAAGA	TTCAGGATGT	TATCTTTTGA
DWV_B_clone11	CTTCTGGTAA	GCGATGGTTG	TTTGATATTG	AATTACAAGA	TTCAGGATGT	TATCTTTTGA
DWV_B_clone12	CTTCTGGTAA	GCGATGGTTG	TTTGATATTG	AATTACAAGA	TTCAGGATGT	TATCTTTTGA
DWV_B_clone13	CTTCTGGTAA	GCGATGGTTG	TTTGATATTG	AATTACAAGA	TTCAGGATGT	TATCTTTTGA
DWV_B_clone14	CTTCTGGTAA	GCGATGGTTG	TTTGATATTG	AATTACAAGA	TTCAGGATGT	TATCTTTTGA
DWV_B_clone15	CTTCTGGTAA	GCGATGGTTG	TTTGATATTG	AATTACAAGA	TTCAGGATGT	TATCTTTTGA
DWV_B_clone16	CTTCTGGTAA	GCGATGGTTG	TTTGATATTG	AATTACAAGA	TTCAGGATGT	TATCTTTTGA
DWV_B_clone17	CTTCTGGTAA	GCGATGGTTG	TTTGATATTG	AATTACAAGA	TTCAGGATGT	TATCTTTTGA
DWV_B_clone18	CTTCTGGTAA	GCGATGGTTG	TTTGATATTG	AATTACAAGA	TTCAGGATGT	TATCTTTTGA
DWV_B_clone19	CTTCTGGTAA	GCGATGGTTG	TTTGATATTG	AATTACAAGA	TTCAGGATGT	TATCTTTTGA
DWV_B_clone20	CTTCTGGTAA	GCGATGGTTG	TTTGATATTG	AATTACAAGA	TTCAGGATGT	TATCTTTTGA
DWV_B_clone21	CTTCTGGTAA	GCGATGGTTG	TTTGATATTG	AATTACAAGA	TTCAGGATGT	TATCTTTTGA
DWV_B_clone22	CTTCTGGTAA	GCGATGGTTG	TTTGATATTG	AATTACAAGA	TTCAGGATGT	TATCTTTTGA
DWV_B_clone23	CTTCTGGTAA	GCGATGGTTG	TTTGATATTG	AATTACAAGA	TTCAGGATGT	TATCTTTTGA
DWV_B_clone24	CTTCTGGTAA	GCGATGGTTG	TTTGATATTG	AATTACAAGA	TTCAGGATGT	TATCTTTTGA
DWV_B_clone25	CTTCTGGTAA	GCGATGGTTG	TTTGATATTG	AATTACAAGA	TTCAGGATGT	TATCTTTTGA
DWV_B_clone26	CTTCTGGCAA	GCGATGGTTG	TTTGATATTG	AATTACAAGA	TTCAGGATGT	TATCTTTTGA
DWV_B_clone27	CTTCTGGTAA	GCGATGGTTG	TTTGATATTG	AATTACAAGA	TTCAGGATGT	TATCTTTTGA
DWV_B_clone28	CTTCTGGTAA	GCGATGGTTG	TTTGATATTG	AATTACAAGA	TTCAGGATGT	TATCTTTTGA
DWV_B_clone29	CTTCTGGTAA	GCGATGGTTG	TTTGATATTG	AATTACAAGA	TTCAGGATGT	TATCTTTTGA
DWV_B_clone30	CTTCTGGTAA	GCGATGGTTG	TTTGATATTG	AATTACAAGA	TTCAGGATGT	TATCTTTTGA
DWV_B_clone31	CTTCTGGTAA	GCGATGGTTG	TTTGATATTG	AATTACAAGA	TTCAGGATGT	TATCTTTTGA
DWV_B_clone32	CTTCTGGTAA	GCGATGGTTG	TTTGATATTG	AATTACAAGA	TTCAGGATGT	TATCTTTTGA
DWV_B_clone33	CTTCTGGTAA	GCGATGGTTG	TTTGATATTG	AATTACAAGA	TTCAGGATGT	TATCTTTTGA
DWV_B_clone34	CTTCTGGTAA	GCGATGGTTG	TTTGATATTG	AATTACAAGA	TTCAGGATGT	TATCTTTTGA
DWV_B_clone35	CTTCTGGTAA	GCGATGGTTG	TTTGATATTG	AATTACAAGA	TTCAGGATGT	TATCTTTTGA
DWV_B_clone36	CTTCTGGTAA	GCGATGGTTG	TTTGATATTG	AATTACAAGA	TTCAGGATGT	TATCTTTTGA
DWV_B_clone37	CTTCTGGTAA	GCGATGGTTG	TTTGATATTG	AATTACAAGA	TTCAGGATGT	TATCTTTTGA
DWV_B_clone38	CTTCTGGTAA	GCGATGGTTG	TTTGATATTG	AATTACAAGA	TTCAGGATGT	TATCTTTTGA
KV_NC_005876	CATCAGGTAA	GCGATGGTTG	TTTGACATTG	AGCTACAAGA	CTCGGGATGT	TATCTCTTGC
DWV_A_NC_004830	CATCAGGYAA	GCGATGGTTG	TTTGAYATTG	AGCTACAAGA	YTCGGGATGT	TATCTCYTGC
DWV_A_clone1	CATCAGGTAA	GCGATGGTTG	TTTGACATTG	AGCTACAAGA	CTCGGGATGT	TATCTCTTGC
DWV_A_clone2	CATCAGGTAA	GCGATGGTTG	TTTGACATTG	AGCTACAAGA	CTCGGGATGT	TATCTCTTGC
DWV_A_clone3	CATCAGGTAA	GCGATGGTTG	TTTGACATTG	AGCTACAAGA	CTCGGGATGT	TATCTCTTGC
DWV_A_clone4	CATCAGGTAA	GCGATGGTTG	TTTGACATTG	AGCTACAAGA	CTCGGGATGT	TATCTCTTGC
DWV_A_clone5	CATCAGGTAA	GCGATGGTTG	TTTGACATTG	AGCTACAAGA	CTCGGGATGT	TATCTCTTGC
DWV_A_clone6	CATCAGGTAA	GCGATGGTTG	TTTGACATTG	AGCTACAAGA	CTCGGGATGT	TATCTCTTGC
DWV_A_clone7	CATCAGGTAA	GCGATGGTTG	TTTGACATTG	AGCTACAAGA	CTCGGGATGT	TATCTCTTGC
DWV_A_clone8	CATCAGGTAA	GCGATGGTTG	TTTGACATTG	AGCTACAAGA	CTCGGGATGT	TATCTCTTGC
DWV_A_clone9	CATCAGGTAA	GCGATGGTTG	TTTGACATTG	AGCTACAAGA	CTCGGGATGT	TATCTCTTGC
DWV_A_clone10	CATCAGGTAA	GCGATGGTTG	TTTGACATTG	AGCTACAAGA	CTCGGGATGT	TATCTCTTGC
DWV_A_clone11	CATCAGGTAA	GCGATGGTTG	TTTGACATTG	AGCTACAAGA	CTCGGGATGT	TATCTCTTGC
DWV_A_clone12	CATCAGGTAA	GCGATGGTTG	TTTGACATTG	AGCTACAAGA	CTCGGGATGT	TATCTCTTGC
DWV_A_clone13	CATCAGGTAA	GCGATGGTTG	TTTGACATTG	AGCTACAAGA	CTCGGGATGT	TATCTCTTGC
DWV_A_clone14	CATCAGGTAA	GCGATGGTTG	TTTGACATTG	AGCTACAAGA	CTCGGGATGT	TATCTCTTGC
DWV_A_clone15	CATCAGGTAA	GCGATGGTTG	TTTGACATTG	AGCTACAAGA	CTCGGGATGT	TATCTCTTGC
DWV_A_clone16	CATCAGGTAA	GCGATGGTTG	TTTGACATTG	AGCTACAAGA	CTCGGGATGT	TATCTCTTGC
DWV_A_clone17	CATCAGGTAA	GCGATGGTTG	TTTGACATTG	AGCTACAAGA	CTCGGGATGT	TATCTCTTGC
DWV_A_clone18	CATCAGGTAA	GCGATGGTTG	TTTGACATTG	AGCTACAAGA	CTCGGGATGT	TATCTCTTGC
DWV_A_clone19	CATCAGGTAA	GCGATGGTTG	TTTGACATTG	AGCTACAAGA	CTCGGGATGT	TATCTCTTGC
DWV_A_clone20	CATCAGGTAA	GCGATGGTTG	TTTGACATTG	AGCTACAAGA	CTCGGGATGT	TATCTCTTGC
DWV_A_clone21	CATCAGGTAA	GCGATGGTTG	TTTGACATTG	AGCTACAAGA	CTCGGGATGT	TATCTCTTGC
DWV_A_clone22	CATCAGGTAA	GCGATGGTTG	TTTGACATTG	AGCTACAAGA	CTCGGGATGT	TATCTCTTGC
DWV_A_clone23	CATCAGGTAA	GCGATGGTTG	TTTGACATTG	AGCTACAAGA	CTCGGGATGT	TATCTCTTGC
DWV_A_clone24	CATCAGGTAA	GCGATGGTTG	TTTGACATTG	AGCTACAAGA	CTCGGGATGT	TATCTCTTGC
DWV_A_clone25	CATCAGGTAA	GCGATGGTTG	TTTGACATTG	AGCTACAAGA	CTCGGGATGT	TATCTCTTGC
DWV_A_clone26	CATCAGGTAA	GCGATGGTTG	TTTGACATTG	AGCTACAAGA	CTCGGGATGT	TATCTCTTGC
DWV_A_clone27	CATCAGGTAA	GCGATGGTTG	TTTGACATTG	AGCTACAAGA	CTCGGGATGT	TATCTCTTGC
DWV_A_clone28	CATCAGGTAA	GCGATGGTTG	TTTGACATTG	AGCTACAAGA	CTCGGGATGT	TATCTCTTGC
DWV_A_clone29	CATCAGGTAA	GCGATGGTTG	TTTGACATTG	AGCTACAAGA	CTCGGGATGT	TATCTCTTGC

Table S3. Cont.

	61			
DWV_B_NC_006494	GAGGGATGAG	ACCTGAACTT	GAGATACAGT	T
DWV_B_clone1	GAGGGATGAG	ACCTGAACTT	GAGATACAGT	T
DWV_B_clone2	GAGGGATGAG	ACCTGAACTT	GAGATACAGT	T
DWV_B_clone3	GAGGGATGAG	ACCTGAACTT	GAGATACAGT	T
DWV_B_clone4	GAGGGATGAG	ACCTGAACTT	GAGATACAGT	T
DWV_B_clone5	GAGGGATGAG	ACCTGAACTT	GAGATACAGT	T
DWV_B_clone6	GAGGGATGAG	ACCTGAACTT	GAGATACAGT	T
DWV_B_clone7	GAGGGATGAG	ACCTGAACTT	GAGATACAGT	T
DWV_B_clone8	GAGGGATGAG	ACCTGAACTT	GAGATACAGT	T
DWV_B_clone9	GAGGGATGAG	ACCTGAACTT	GAGATACAGT	T
DWV_B_clone10	GAGGGATGAG	ACCTGAACTT	GAGATACAGT	T
DWV_B_clone11	GAGGGATGAG	ACCTGAACTT	GAGATACAGT	T
DWV_B_clone12	GAGGGATGAG	ACCTGAACTT	GAGATACAGT	T
DWV_B_clone13	GAGGGATGAG	ACCTGAACTT	GAGATACAGT	T
DWV_B_clone14	GAGGGATGAG	ACCTGAACTT	GAGATACAGT	T
DWV_B_clone15	GAGGGATGAG	ACCTGAACTT	GAGATACAGT	T
DWV_B_clone16	GAGGGATGAG	ACCTGAACTT	GAGATACAGT	T
DWV_B_clone17	GAGGGATGAG	ACCTGAACTT	GAGATACAGT	T
DWV_B_clone18	GAGGGATGAG	ACCTGAACTT	GAGATACAGT	T
DWV_B_clone19	GAGGGATGAG	ACCTGAACTT	GAGATACAGT	T
DWV_B_clone20	GAGGGATGAG	ACCTGAACTT	GAGATACAGT	T
DWV_B_clone21	GAGGGATGAG	ACCTGAACTT	GAGATACAGT	T
DWV_B_clone22	GAGGGATGAG	ACCTGAACTT	GAGATACAGT	T
DWV_B_clone23	GAGGGATGAG	ACCTGAACTT	GAGATACAGT	T
DWV_B_clone24	GAGGGATGAG	ACCTGAACTT	GAGATACAGT	T
DWV_B_clone25	GAGGGATGAG	ACCTGAACTT	GAGATACAGT	T
DWV_B_clone26	GAGGGATGAG	ACCTGAACTC	GAGATACAGT	T
DWV_B_clone27	GAGGGATGAG	ACCTGAACTT	GAGATACAGT	T
DWV_B_clone28	GAGGGATGAG	ACCTGAACTT	GAGATACAGT	T
DWV_B_clone29	GAGGGATGAG	ACCTGAACTT	GAGATACAGT	T
DWV_B_clone30	GAGGGATGAG	ACCTGAACTT	GAGATACAGT	T
DWV_B_clone31	GAGGGATGAG	ACCTGAACTT	GAGATACAGT	T
DWV_B_clone32	GAGGGATGAG	ACCTGAACTT	GAGATACAGT	T
DWV_B_clone33	GAGGGATGAG	ACCTGAACTT	GAGATACAGT	T
DWV_B_clone34	GAGGGATGAG	ACCTGAACTT	GAGATACAGT	T
DWV_B_clone35	GAGGGATGAG	ACCTGAACTT	GAGATACAGT	T
DWV_B_clone36	GAGGGATGAG	ACCTGAACTT	GAGATACAGT	T
DWV_B_clone37	GAGGGATGAG	ACCTGAACTT	GAGATACAGT	T
DWV_B_clone38	GAGGGATGAG	ACCTGAACTT	GAGATACAGT	T
KV_NC_005876	GTGGAATGCG	TCCCGAACTT	GAGATTCAAT	T
DWV_A_NC_004830	GTGGAATGCG	TCCCGAACTT	GAGATTCAAT	T
DWV_A_clone1	GTGGAATGCG	TCCCGAACTT	GAGATTCAAT	T
DWV_A_clone2	GTGGAATGCG	TCCCGAACTT	GAGATTCAAT	T
DWV_A_clone3	GTGGAATGCG	TCCCGAACTT	GAGATTCAAT	T
DWV_A_clone4	GTGGAATGCG	TCCCGAACTT	GAGATTCAAT	T
DWV_A_clone5	GTGGAATGCG	TCCCGAACTT	GAGATTCAAT	T
DWV_A_clone6	GTGGAATGCG	TCCCGAACTT	GAGATTCAAT	T
DWV_A_clone7	GTGGAATGCG	TCCCGAACTT	GAGATTCAAT	T
DWV_A_clone8	GTGGAATGCG	TCCCGAACTT	GAGATTCAAT	T
DWV_A_clone9	GTGGAATGCG	TCCCGAACTT	GAGATTCAAT	T
DWV_A_clone10	GTGGAATGCG	TCCCGAACTT	GAGATTCAAT	T
DWV_A_clone11	GTGGAATGCG	TCCCGAACTT	GAGATTCAAT	T
DWV_A_clone12	GTGGAATGCG	TCCCGAACTT	GAGATTCAAT	T
DWV_A_clone13	GTGGAATGCG	TCCCGAACTT	GAGATTCAAT	T
DWV_A_clone14	GTGGAATGCG	TCCCGAACTT	GAGATTCAAT	T
DWV_A_clone15	GTGGAATGCG	TCCCGAACTT	GAGATTCAAT	T
DWV_A_clone16	GTGGAATGCG	TCCCGAACTT	GAGATTCAAT	T
DWV_A_clone17	GTGGAATGCG	TCCCGAACTT	GAGATTCAAT	T
DWV_A_clone18	GTGGAATGCG	TCCCGAACTT	GAGATTCAAT	T
DWV_A_clone19	GTGGAATGCG	TCCCGAACTT	GAGATTCAAT	T
DWV_A_clone20	GTGGAATGCG	TCCCGAACTT	GAGATTCAAT	T
DWV_A_clone21	GTGGAATGCG	TCCCGAACTT	GAGATTCAAT	T
DWV_A_clone22	GTGGAATGCG	TCCCGAACTT	GAGATTCAAT	T
DWV_A_clone23	GTGGAATGCG	TCCCGAACTT	GAGATTCAAT	T
DWV_A_clone24	GTGGAATGCG	TCCCGAACTT	GAGATTCAAT	T
DWV_A_clone25	GTGGAATGCG	TCCCGAACTT	GAGATTCAAT	T
DWV_A_clone26	GTGGAATGCG	TCCCGAACTT	GAGATTCAAT	T
DWV_A_clone27	GTGGAATGCG	TCCCGAACTT	GAGATTCAAT	T
DWV_A_clone28	GTGGAATGCG	TCCCGAACTT	GAGATTCAAT	T
DWV_A_clone29	GTGGAATGCG	TCCCGAACTT	GAGATTCAAT	T

Table S4. List of primers used in this study.

Primer Target	Name	Sequence	Application	Reference
DWV-A (RdRp)	DWV-F2	TGTCTTCATTAAAGCCACCTGGAA	qPCR	[1]
DWV-A (RdRp)	DWV-R2a	TTTCCTCATTAACTGTGTCGTTGAT	qPCR	"
DWV-B (RdRp)	VDV-F2	TATCTTCATTAAAACCGCCAGGCT	qPCR	"
DWV-B (RdRp)	VDV-R2a	CTTCCTCATTAACTGAGTTGTTGTC	qPCR	"
DWV-A (RdRp)	F8668	TTCATTAAAGCCACCTGGAACATC	qPCR	[2]
DWV-A (RdRp)	B8757	TTTCCTCATTAACTGTGTCGTTGA	qPCR	"
DWV-A (RdRp)	DWV-F1a	GGAAACATCTGGAATTAGCGACAAA	Stand' curve	[1]
DWV-B (RdRp)	VDV-F1a	GAAAACATTTGGAATTAGCAACGAC	Stand' curve	"
DWV-A / -B (RdRp)	DWVDV-7A-R	AATCCGTGAATATAGTGTGAGG	Stand' curve	"
SBPV	SPV-F3177	GCGCTTTAGTTCAATTGCC	qPCR	[3]
SBPV	SPV-B3363	ATTATAGGACGTGAAAATATAC	qPCR	"
SBPV	SBPV-var-F2	GTGCTTTAGTTCAATTACCATTG	qPCR	This study
SBPV	SBPV-var-R	ATTATGGGACGTGAGAAT ATAC	qPCR	This study
ABPV	ABPV-F6548	TCATACCTGCCGATCAAG	qPCR	[4]
IAPV	IAPV-F6627	CCATGCCTGGCGATTCAC	qPCR	"
ABPV/IAPV	KIABPV-B6707	CTGAATAATACTGTGCGTATC	qPCR	"
BQCV	BQCV-F7893	AGTGGCGGAGATGTATGC	qPCR	[1]
BQCV	BQCV-B8150	GGAGGTGAAGTGGCTATATC	qPCR	"
SBV	SBV-F3164	TTGGAACTACGCATTCTCTG	qPCR	"
SBV	SBV-B3461	GCTCTAACCTCGCATCAAC	qPCR	"
RP49	RP49-qF	AAGTTCATTCGTCACCAGAG	qPCR	[2]
RP49	RP49-qB	CTTCCAGTTCCTTGACATTATG	qPCR	"

Table S4. References

1 McMahon, D.P., Fuerst, M.A., Caspar, J., Theodorou, P., Brown, M.J.F., Paxton, R.J. A sting in the spit: widespread cross-infection of multiple RNA viruses across wild and managed bees. J Anim Ecol 83, doi:10.1111/1365-2656.12345 (2015).

2 Forsgren, E., de Miranda, J.R., Isaksson, M., Wei, S., and Fries, I. Deformed wing virus associated with Tropilaelaps mercedesae infesting European honey bees (Apis mellifera). Exp Appl Acarol 47, 87-97 (2009).

3 de Miranda, J.R. et al. Genetic characterization of slow bee paralysis virus of the honeybee (Apis mellifera L.). J Gen Virol 91, 2524-2530 (2010).

4 de Miranda, J.R., Cordoni, G., and Budge, G. The Acute bee paralysis virus-Kashmir bee virus-Israeli acute paralysis virus complex. J Invertebr Pathol 103

Table S5. Description of adjusted BEEHAVE model parameters. Modified values are emphasized in underlined italics.

Model scenario (1) DWV-A.

**Parameter**	**Parameter value**	**Default value**	**Description**
MORTALITY_INHIVE	0.004	0.004	Daily mortality rate of healthy in-hive bees and foragers
MORTALITY_INHIVE_INFECTED_AS_ADULT	0.012	0.012	Daily mortality rate of in-hive bees and foragers, infected as adults
MORTALITY_INHIVE_INFECTED_AS_PUPAE	0.012	0.012	Daily mortality rate of in-hive bees and foragers, infected as pupae

Model scenario (2) DWV-B

**Parameter**	**Parameter value**	**Default value**	**Description**
MORTALITY_INHIVE	0.004	0.004	Daily mortality rate of healthy in-hive bees and foragers
MORTALITY_INHIVE_INFECTED_AS_ADULT	*0.016*	0.012	Daily mortality rate of in-hive bees and foragers, infected as adults
MORTALITY_INHIVE_INFECTED_AS_PUPAE	*0.016*	0.012	Daily mortality rate of in-hive bees and foragers, infected as pupae

Conservative model scenario (2) DWV-B

**Parameter**	**Parameter value**	**Default value**	**Description**
MORTALITY_INHIVE	0.004	0.004	Daily mortality rate of healthy in-hive bees and foragers
MORTALITY_INHIVE_INFECTED_AS_ADULT	*0.016*	0.012	Daily mortality rate of in-hive bees and foragers, infected as adults
MORTALITY_INHIVE_INFECTED_AS_PUPAE	0.012	0.012	Daily mortality rate of in-hive bees and foragers, infected as pupae

Table S6. Final Cox proportional hazard model of cage mortality following experimental inoculation, from day 13 p.i. onwards. C, control; A, DWV-A; B, DWV-B; M, mixed DWV-A and -B. SE, Standard error; SD, Standard deviation. a Equivalent to the hazard ratio, the instantaneous risk of death for bees in each treatment compared with the baseline treatment level (in this case C). Higher levels of β indicate higher risk of death.

Parameters	Coefficients			Model testing		
	β	SE (β)	Exp (β) [a]	χ^2 (LRT)	df	*P*-value
Fixed variable						
Treatment				43.102	3	<0.00001*
C	0	-	1			
A	4.742	0.671	114.642			
B	6.464	0.708	641.819			
M	6.061	0.698	428.914			
Random variable	SD	Variance				
Cage	0.542	0.293				
